# Organ ischemia during partial resuscitative endovascular balloon occlusion of the aorta: Dynamic 4D Computed tomography in swine

**DOI:** 10.1038/s41598-020-62582-y

**Published:** 2020-03-30

**Authors:** Yosuke Matsumura, Akiko Higashi, Yoshimitsu Izawa, Shuji Hishikawa, Hiroshi Kondo, Viktor Reva, Shigeto Oda, Junichi Matsumoto

**Affiliations:** 1 Department of Emergency and Critical Care Medicine, Chiba University Graduate School of Medicine, Chiba, Chiba Japan; 2 Department of Emergency and Critical Care Medicine, Saiseikai Utsunomiya Hospital, Utsunomiya, Tochigi Japan; 3 Department of Emergency and Critical Care Medicine, Jichi Medical University, Shimotsuke, Tochigi Japan; 4 Center for Development of Advanced Medical Technology, Jichi Medical University, Shimotsuke, Tochigi Japan; 5 Department of Radiology, Teikyo University School of Medicine, Itabashi, Tokyo Japan; 6 Department of War Surgery, Kirov Military Medical Academy, Ulitsa Akademika Lebedeva, St Petersburg, Russia; 7 Department of Emergency and Critical Care Medicine, St. Marianna University School of Medicine, Kawasaki, Kanagawa Japan

**Keywords:** Experimental models of disease, Translational research

## Abstract

Resuscitative endovascular balloon occlusion of the aorta (REBOA) increases proximal pressure, and simultaneously induces distal ischemia. We aimed to evaluate organ ischemia during partial REBOA (P-REBOA) with computed tomography (CT) perfusion in a swine model. The maximum balloon volume was recorded as total REBOA when the distal pulse pressure ceased. The animals (n = 4) were scanned at each 20% of the maximum balloon volume, and time-density curve (TDC) were analysed at the aorta, portal vein (PV), liver parenchyma, and superior mesenteric vein (SMV, indicating mesenteric perfusion). The area under the TDC (AUTDC), the time to peak (TTP), and four-dimensional volume-rendering images (4D-VR) were evaluated. The TDC of the both upper and lower aorta showed an increased peak and delayed TTP. The TDC of the PV, liver, and SMV showed a decreased peak and delayed TTP. The dynamic 4D-CT analysis suggested that organ perfusion changes according to balloon volume. The AUTDC at the PV, liver, and SMV decreased linearly with balloon inflation percentage to the maximum volume. 4D-VR demonstrated the delay of the washout in the aorta and retrograde flow at the inferior vena cava in the highly occluded status.

## Introduction

Resuscitative endovascular balloon occlusion of the aorta (REBOA) has been recently accepted as a feasible method of resuscitation in patients with refractory haemorrhagic shock^1–3^. REBOA increases proximal blood pressure through the minimally invasive aortic occlusion^4^. However, it induces distal ischaemia of the visceral organs and lower extremities, which causes inflammatory sequelae^5^ and may be life threatening or limb threatening^6–8^. Thus, investigating organ ischaemia during REBOA is essential, and a better understanding may contribute to the safer resuscitation of patients with haemorrhagic shock.

Currently, partial REBOA (P-REBOA) is believed to mitigate distal ischaemia and to extend survival^9–12^. To evaluate the degree of P-REBOA, computed tomography (CT) imaging has been employed in a swine model^13–15^. The widest cross-sectional area of the balloon and the proximal or distal pressure were also used to define the degree of P-REBOA^16^.

However, despite recent studies, the association between the degree of P-REBOA and distal organ ischaemia has not been evaluated yet. Proximal and distal pressures are clinically measurable; however, arterial pressure may not necessarily indicate blood flow or organ ischaemia. To avoid organ dysfunction, organ ischaemia during REBOA should be investigated.

Recent advancements in CT scan technology have enabled visualising organ ischaemia, and this modality has been utilised in cerebral, myocardial, or visceral organs^17–20^. The objective of this study was to investigate organ ischaemia during staged P-REBOA. We analysed the time-density curve (TDC) of dynamic four dimensional (4D)-CT in a swine model, which has similar anatomy to human, and investigated the correlation of inflated balloon volume and organ ischaemia or visceral blood flow.

## Materials and methods

### Overview

This study was conducted in an accredited animal research laboratory (Center for Development of Advanced Medical Technology, Jichi Medical University, Tochigi, Japan). Approval was obtained from the Animal Experiment Committee at Center for Experimental Medicine, Jichi Medical University prior to conducting the study (authorisation no. 17045-01). All methods were carried out in accordance with relevant guidelines and regulations. Healthy female non-pregnant domestic pigs (n = 5) were obtained from Sanesu Breeding Co., Ltd. (Chiba, Japan) and used in the present study. The first animal was used for model verification to minimise the technical error and effects of subjective bias, and the remaining 4 animals were used for analysis. Previous animal study^16^ used six subjects for evaluation of the partial REBOA. We evaluated the results of first four animals, which had acceptable reproducibility by investigators’ assessment. Thus we did not add the further experimental animals by the “Reduction” principle in the animal welfare. The animals were quarantined for a minimum of 7 days and fasted for 24 h with free access to water before enrolment in the experimental protocol. At the time of experimentation, the animals were between 3 and 4 months of age and weighed 35–45 kg. The experimental protocol included two phases: animal preparation and dynamic 4D-CT scan with P-REBOA, followed by analysis of CT data.

### Animal preparation

The animals were premedicated in the intramuscularly with 0.06 mg/kg medetomidine (Nippon Zenyaku Kogyo Co., Ltd., Fukushima, Japan), 0.3 mg/kg midazolam (Astellas Pharma Inc., Tokyo, Japan), and 0.08 mg/kg atropine (Mitsubishi Tanabe Pharma Corporation, Osaka, Japan) at 9 am in the animal cage. After confirmation of sedation and endotracheal intubation in the animal operation room, maintenance anaesthesia consisting of 3% sevoflurane was induced and 1% propofol was injected intravenously as needed (Maruishi Pharmaceutical Co. Ltd, Osaka, Japan). The animals were mechanically ventilated with tidal volumes of 7–10 mL/kg and a respiratory rate of 10–15 breaths/min, which were sufficient to maintain the end-tidal CO_2_ at 40 ± 5 mmHg. The pigs were placed on a warming blanket set at 39 °C to maintain body temperature.

### Surgical procedures and REBOA placement

**Figure 1:**
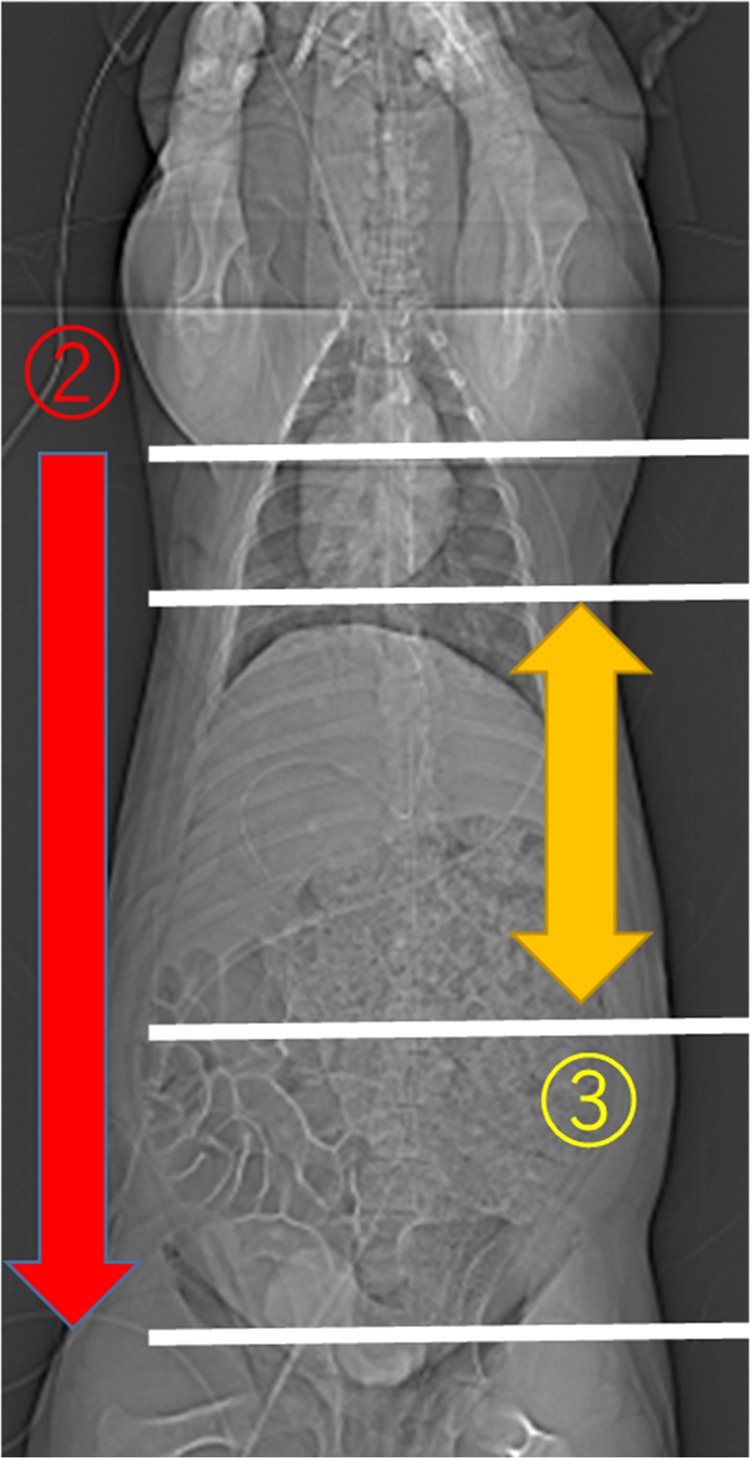
Scanning protocol (1) Scout view. Balloon positioning. (2) Pre-perfusion scan. Confirmation of the position of the inflated balloon. Non-enhanced, 3-mm slice. Scan range: from the supra-diaphragm to the entire abdomen. (3) Computed tomography perfusion. Blood flow and organ ischaemia. 3-mm slice Temporal resolution, 2 s + 4 s (multiple), 49 phases. Scan range: 204 mm from the top of the liver (69 slices). Tube potential, 120 kV; Tube current, 320 mA; Rotation time, 0.3 sec.

After general anaesthesia induction, the right neck was exposed, and an arterial line was catheterised for proximal pressure monitoring and blood sampling into the right carotid artery. A central venous catheter was placed into the right jugular vein. Both groins were exposed, and a 9-Fr sheath was placed into the right femoral artery for insertion of a 7-Fr REBOA catheter (Rescue Balloon^®^; Tokai Medical Products, Aichi, Japan). Then, an arterial line was placed into the left femoral artery for distal pressure monitoring. A large sheath size was chosen to ensure the atraumatic removal of the balloon catheter, allowing for repeated use. Heparinised saline was used only for flushing of sheaths, and no systemic anticoagulation was administered. Acetated Ringer’s solution was infused to ensure that the stroke volume variation was 13%. The animals were transferred to the CT scanner (SOMATOM^®^ Definition AS + [128-slice]; Siemens Healthcare GmbH, Erlangen, Germany) while maintaining general anaesthesia. A REBOA catheter was deployed into the thoracic aorta, with the balloon position maintained above the diaphragm level with a CT scout view. The REBOA catheter was fixed to the skin, and the balloon was gradually inflated with close monitoring of distal pressure. Total REBOA was defined as the complete cessation of the distal pulse pressure, and the maximum balloon volume at total REBOA was documented in each animal described in our previous study^21^. At the total REBOA inflation, increased proximal pressure was confirmed, and the catheter position was maintained, preventing downstream migration (Fig. 1).

### Dynamic 4D-CT scan

To evaluate the changes in organ perfusion according to the degree of P-REBOA, the animals were scanned by changing every 20% of the maximum balloon volume. A scan cycle included a pre-perfusion scan and a dynamic 4D-CT scan (Fig. 1). The pre-perfusion scan obtained a non-enhanced image by scanning from the supra-diaphragm level to the entire abdomen to confirm the position of the inflated balloon. For the dynamic 4D-CT, a 600 mg iodine/kg bolus of iopamidol (300 mg iodine/mL Iopaque^®^; FujiPharma, Tokyo, Japan) was administered intravenously in 30 s followed by flushing with 10 mL of 0.9% saline through the right jugular vein. The dynamic 4D-CT scan was initiated at the beginning of contrast injection followed by 48 phases (2 s, 40 phases; 4 s, 8 phases) of scanning with multiple temporal resolutions, including a scan range of 204 mm from the top of the liver. Acetated Ringer’s solution was infused continuously to wash out the contrast material between every scan cycle of approximately 30 min. The 4D-volume rendering (VR) images were generated from the CT data to visualize the blood flow and solid organ enhancement. The images were reconstructed and analyzed with a 3D workstation (Ziostation2® PLUS Classic S, Ziosoft Inc., Tokyo, Japan), then combined with Apowersoft video converter V4.8.4.23 (Apowersoft Ltd, Hong Kong).

### Analysis of the TDC

The dynamic 4D-CT data were analysed at three different scan levels. The section where the upper aorta, inferior vena cava (IVC), right hepatic vein (RHV), portal vein (PV), and liver parenchyma are all displayed in a cross-sectional view was defined as the upper section. The middle section was chosen for the TDC of SMV. The SMV merges with splenic vein and becomes PV (merge point). The middle section was defined as the slice at 6 mm caudal side from the merge point. The lower section is the most distal scan range (approximately 13 cm distal from the upper section) (Supplement 1). The upper aorta, IVC, RHV, PV, and liver parenchyma were analysed on the upper section by a region of interest (ROI). The SMV was evaluated on the middle section, and the lower aorta was analysed on the lower section, respectively. We drew the TDCs for the evaluation of blood flow and organ ischaemia. The TDC was calculated using the elevation from the baseline, because the baseline density at each scan was not the same, and was different among each subject. The PV or liver was evaluated as an indicator of the liver perfusion, and the SMV was evaluated as an indicator of the mesenteric perfusion. The TDC was plotted, and the time to peak (TTP) was determined in each region of interest.

The area under the TDC (AUTDC) was calculated as a primary analysis to evaluate the changes in perfusion caused by changes in the degree of occlusion. The change rate of the AUTDC was assessed as a secondary analysis based on the non-occluded status (0% occlusion) as a reference. Density was described in Hounsfield units (HU), and time was described in seconds (s). The degree of P-REBOA and the percentage changes in the AUC of the TDC were analysed with linear regression using GraphPad Prism 6.07 for Windows (GraphPad Software Inc., La Jolla, CA, USA).

### Ethical approval

This study was conducted in an accredited animal research laboratory (Centre for Development of Advanced Medical Technology, Jichi Medical University, Tochigi, Japan). Institutional Animal Experiment Committee approval was obtained before beginning the study (authorization number 17045–01).

## Results

All experimental animals were anaesthetised and underwent surgical procedures safely. No adverse event was observed throughout the entire experiment. Four animals were analysed, the length was 107.3 ± 5.9 cm, and the weight was 37.8 ± 4.2 kg.

**Figure 2:**
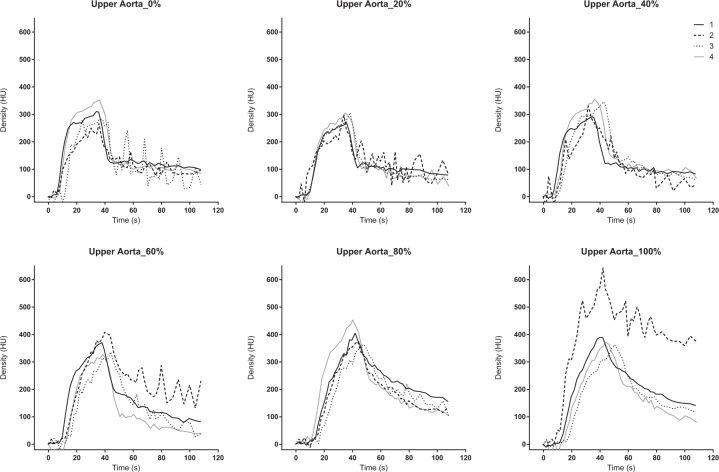
Time-density curve in the upper aorta under a regulated occlusion volume in partial resuscitative endovascular balloon occlusion of the aorta. The peak density of the time-density curve increased and the time to peak was delayed as the balloon volume increased.

**Figure 3:**
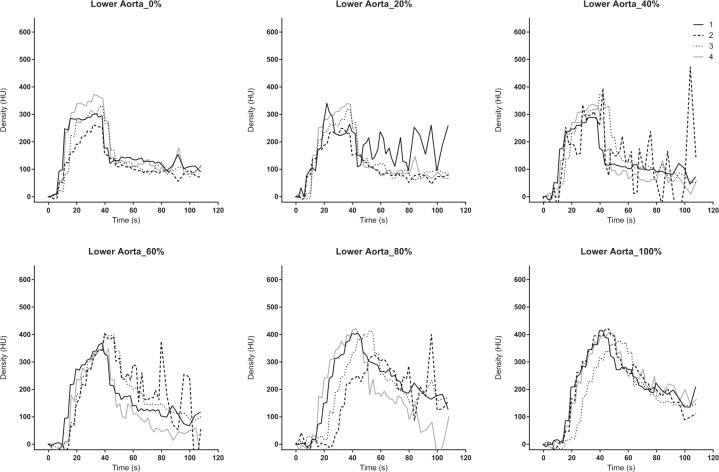
Time-density curve in the lower aorta under a regulated occlusion volume in partial resuscitative endovascular balloon occlusion of the aorta. The peak density of the time-density curve increased and the time to peak was delayed as the balloon volume increased.

The 4D-VR images demonstrated entire blood flow and organ perfusion (Video). The peak density in the aorta increased, and the TTP was delayed according to the inflation of the balloon. The density was more increased in the upper aorta but demonstrated the same trend. (Figs. 2 and 3). The TTP was delayed at the balloon volume of ≥40% (Table 1), and the peak density was increased according to the balloon inflation (Table 2). The contrast in the aorta was washed out rapidly in the 0% but remained at the end of the scan in the 100% (Video).

**Table 1:** Changes in the time to peak according to degree of P-REBOA.

	0%	20%	40%	60%	80%	100%
**Upper aorta**						
1	34.4	36.0	34.4	38.4	42.4	42.4
2	36.0	34.7	32.0	40.0	44.0	42.7
3	38.4	38.4	42.4	44.0	48.0	50.5
4	36.0	34.4	36.0	38.4	40.0	44.0
**Lower aorta**						
1	34.4	22.4	36.0	38.4	44.0	42.4
2	32.0	32.0	42.7	40.0	56.0	46.7
3	38.4	36.0	40.0	40.0	52.0	54.5
4	32.0	36.0	34.4	42.4	42.4	46.5
**IVC**						
1	40.0	52.0	48.0	60.0	68.0	62.4
2	40.0	44.0	72.0	88.1	38.7	34.7
3	40.0	48.0	56.0	74.5	36.0	40.0
4	48.0	46.5	48.0	54.5	108.1	32.0
**RHV**						
1	58.4	70.4	78.5	108.1	72.1	72.1
2	88.1	96.1	108.1	74.7	38.7	36.0
3	88.1	104.1	108.1	38.4	34.4	42.4
4	80.0	78.5	104.1	104.1	84.1	40.0
**PV**						
1	46.4	52.0	50.4	100.1	56.0	108.1
2	44.0	56.0	68.0	92.1	80.0	20.0
3	52.0	60.0	96.1	74.5	104.1	22.4
4	52.0	52.0	60.0	72.0	108.1	74.5
**Liver**						
1	58.4	66.4	70.4	104.1	108.1	7.9
2	76.0	88.1	92.1	74.7	92.1	15.9
3	70.5	104.1	108.1	96.1	62.5	60.0
4	70.5	64.0	92.1	104.1	104.1	78.5
**SMV**						
1	44.1	48.8	48.8	104.1	72.1	88.1
2	53.3	49.3	76.1	108.1	13.2	4.0
3	61.4	60.1	72.1	80.1	13.3	61.4
4	48.1	48.9	56.9	76.1	104.1	88.1

Data are mean ± standard deviation in seconds.

P-REBOA, partial resuscitative endovascular balloon occlusion of the aorta; PV, portal vein; SMV, superior mesenteric vein.

**Table 2:** Changes in the peak value in the time density curve according to degree of P-REBOA.

	0%	20%	40%	60%	80%	100%
**Upper aorta**						
1	310.2	273.6	290.4	370.5	405.4	389.2
2	266.6	296.5	330.1	407.9	374.6	642.8
3	282.2	306.2	345.4	336.0	362.8	363.2
4	352.3	303.8	355.5	327.2	453.4	371.7
**Lower aorta**						
1	301.8	341.4	289.4	369.4	405.0	414.6
2	262.1	249.3	335.5	404.5	325.3	420.5
3	330.7	317.9	374.0	403.3	414.2	377.0
4	372.8	339.8	336.8	348.8	421.1	407.1
**IVC**						
1	175.5	90.3	119.1	83.0	94.0	90.2
2	157.3	129.9	103.6	68.4	302.9	169.4
3	179.6	189.8	159.0	35.9	404.8	488.8
4	224.1	177.8	199.1	160.7	90.4	40.0
**RHV**						
1	163.2	138.6	122.3	43.6	86.5	77.7
2	110.2	112.0	57.9	19.6	273.9	178.5
3	111.6	93.2	61.0	45.3	384.1	341.0
4	159.9	125.0	116.5	87.3	62.7	31.3
**PV**						
1	176.5	130.2	146.5	103.5	15.1	5.2
2	137.1	144.6	103.4	14.3	31.5	5.8
3	127.5	104.1	98.1	29.7	11.2	7.2
4	190.4	186.1	165.6	136.5	111.4	27.8
**Liver**						
1	83.8	67.1	72.1	42.7	2.0	15.0
2	60.8	60.5	50.9	13.4	9.1	7.1
3	68.4	55.5	53.6	10.2	27.3	33.5
4	78.2	68.4	71.6	59.4	19.7	6.9
**SMV**						
1	195.8	143.3	141.5	111.2	8.8	9.8
2	143.1	157.6	132.2	22.7	10.9	20.1
3	157.6	106.7	109.4	37.0	30.2	38.0
4	207.9	182.7	162.4	148.0	104.8	23.4

Data are mean ± standard deviation in Hounsfield units.

P-REBOA, partial resuscitative endovascular balloon occlusion of the aorta; PV, portal vein; SMV, superior mesenteric vein.

**Figure 4:**
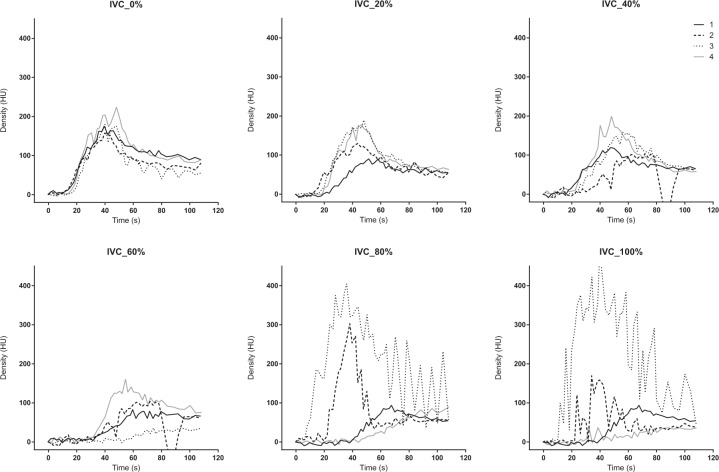
Time-density curve in the inferior vena cava under a regulated occlusion volume in partial resuscitative endovascular balloon occlusion of the aorta. The peak density the time-density curve decreased gradually and the time to peak was delayed as the balloon volume increased until 60%. Two individuals demonstrated sudden increased enhancement at the analyzed level.

**Figure 5:**
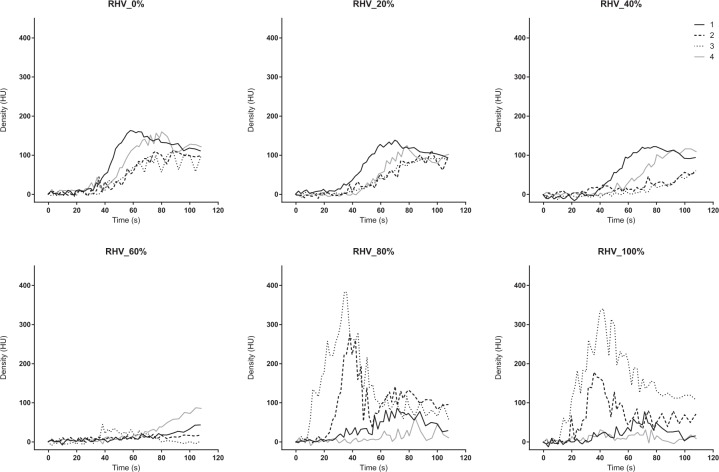
Time-density curve in the right hepatic vein under a regulated occlusion volume in partial resuscitative endovascular balloon occlusion of the aorta. The peak density the time-density curve decreased gradually and the time to peak was delayed as the balloon volume increased until 60%. Two individuals demonstrated sudden increased enhancement at the analyzed level.

The TDC of the IVC and RHV showed irregular patterns. The peak density at the IVC and RHV decreased, and the TTP was delayed during the non-occluded or less occluded condition (0–60%). Then, the peak suddenly increased at 80% or more (Figs. 4 and 5). The TTP became shorter at 80% and 100% occlusion (Table 1). The 4D-VR images demonstrated that the contrast in the IVC came from the caudal side in the 0 to 40% occlusion, while it came from the cranial side in the 80 and 100% occlusion (Video).

**Figure 6:**
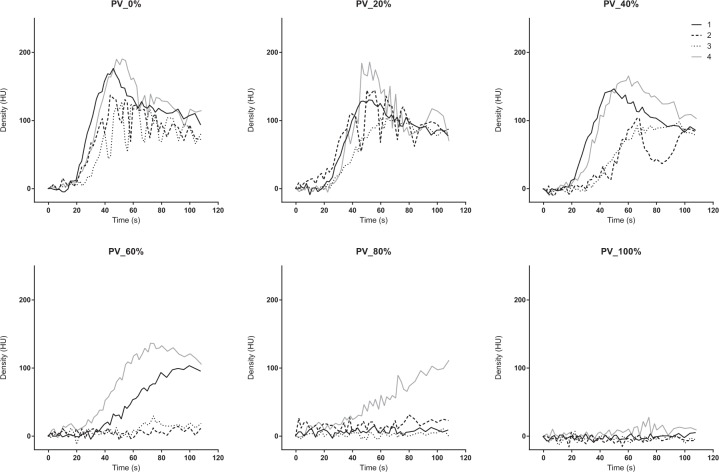
Time-density curve in the portal vein under a regulated occlusion volume in partial resuscitative endovascular balloon occlusion of the aorta. The peak density the time-density curve decreased gradually and the time to peak was delayed as the balloon volume increased.

**Figure 7:**
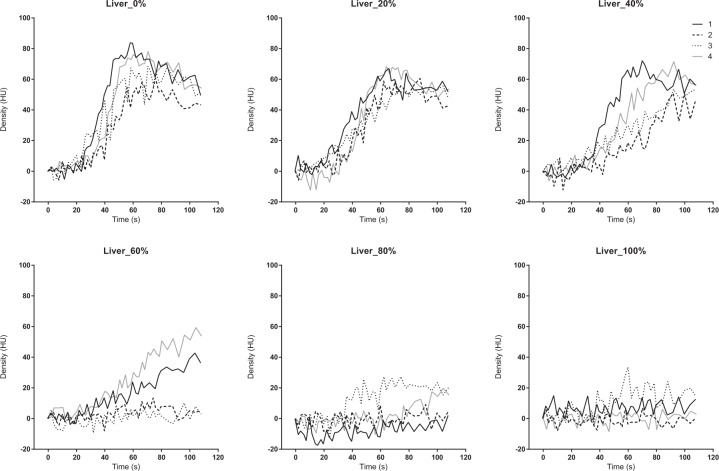
Time-density curve in the liver parenchyma under a regulated occlusion volume in partial resuscitative endovascular balloon occlusion of the aorta. The peak density the time-density curve decreased gradually and the time to peak was delayed as the balloon volume increased.

**Figure 8:**
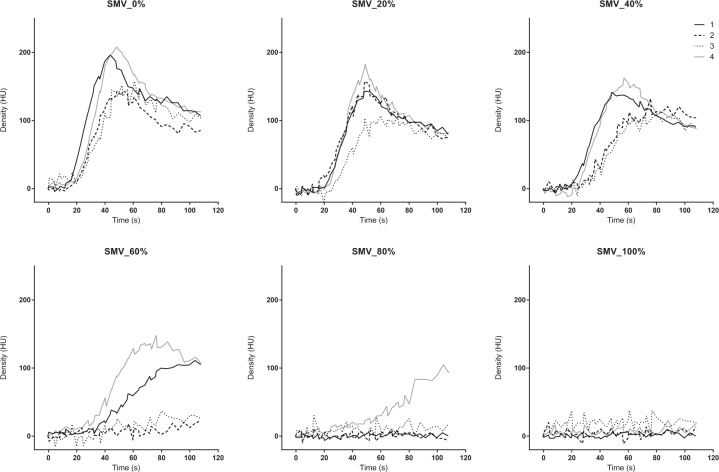
Time-density curve in the superior mesenteric vein a regulated occlusion volume in partial resuscitative endovascular balloon occlusion of the aorta. The peak density the time-density curve decreased gradually and the time to peak was delayed as the balloon volume increased.

**Figure 9:**
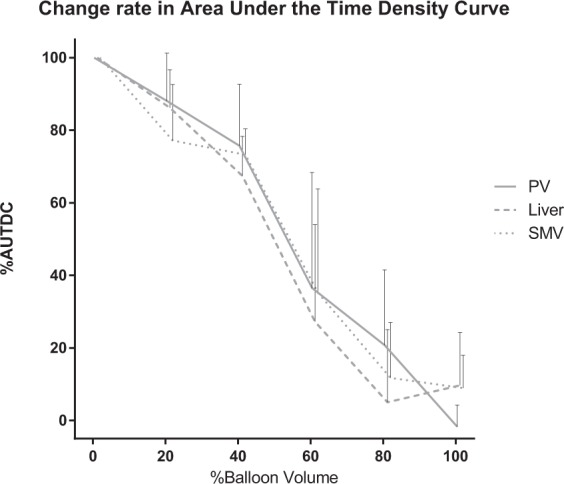
The Change rate of the area under the time-density curve (AUTDC) under regulated occlusion volume in partial resuscitative endovascular balloon occlusion of the aorta. The AUTDC of the aorta increased as the occlusion volume increased (at ≥60% occlusion) (Y = 0.730*X + 81.9, r^2^ = 0.810, P = 0.015). The AUTDC of the portal vein (PV), liver parenchyma, and superior mesenteric vein (SMV) decreased linearly from 0% to 100% (PV, Y = −1.071*X + 106.8, r^2^ = 0.972, P = 0.0003; liver, Y = −1.050*X + 101.8, r^2^ = 0.933, P = 0.0017; SMV, Y = −0.985*X + 100.3, r^2^ = 0.952, P = 0.0009). Video. Four dimensional-volume rendering images of the dynamic computed tomography data. The degree of occlusion increases from 0% (upper left) to 100% (lower right). The contrast in the aorta was washed out rapidly in the 0% but remains at the end of the scan in the 100%. The contrast in the inferior vena cava (IVC) came from the caudal side in the 0 to 40% but came from the cranial side in 80 and 100%. The IVC and hepatic vein were enhanced by the back flow in 80 and 100%.

The TDC of the PV, liver parenchyma, and SMV presented similar patterns. The peak density decreased and the TTP was delayed as the occlusion degree increased from 0% to 80%. The TDC did not show a clear peak density at 100% occlusion (Figs. 6, 7 and 8). The AUTDC of the PV or liver, and that of the SMV decreased linearly as the occlusion volume increased (PV, Y = −1.071*X + 106.8, r^2^ = 0.972, P = 0.0003; liver, Y = −1.050*X + 101.8, r^2^ = 0.933, P = 0.0017; SMV, Y = −0.985*X + 100.3, r^2^ = 0.952, P = 0.0009) (Fig. 9).

## Discussion

This is the first study to demonstrate blood flow and organ ischaemia during P-REBOA by analysing TDC of dynamic 4D-CT. The TDC of both upper and lower aorta showed an elevated peak, delayed TTP, and increased AUTDC as the occlusion degree increased. The TDC of the PV or liver, and that of the SMV demonstrated a declined peak, delayed TTP, and linearly decreased AUTDC as the occlusion degree increased.

Interestingly, the TDC of the aorta showed a higher peak density, delayed TTP, and increased AUC with increased balloon inflation volume. This trend was sharply observed at the upper aorta than the lower aorta. Although the aortic blood flow and distal pressure decreased, the peak density increased even when the balloon volume was at the maximum, which was predefined as the cessation of distal pulse pressure. It is speculated that the balloon and aortic wall were not watertight, and blood and the contrast flowed distally even when the distal pressure ceased. The 4D-VR images clearly visualized that aortic balloon itself interrupted to wash-out the contrast in the aorta; thus, the contrast material remained in the aorta in the highly occluded status. As a result, the high degree aortic occlusion led augmented and prolonged enhancement, which could explain the increased peak and AUTDC. Complete blockade of aortic flow without any enhancement of the distal aorta may require overinflation after the cessation of the distal pulse pressure.

The TDC of the IVC and RHV showed a sudden change in the curve pattern at high degrees of inflation. The peak density and AUTDC gradually decreased from 0% to 60% occlusion, then suddenly increased at 80%. The TTP came earlier at ≥80% occlusion. This sudden change at 80% could be explained by contrast overflow to the IVC from the SVC. The 4D-VR images visualized the retrograde flow in the IVC from the cranial side, which also caused the sudden changes of the TDCs in the IVC or RHV. The flow of the SVC was accelerated, and that of the IVC was decelerated by caval congestion and elevated aortic afterload. This phenomenon presumably indicated that a high degree (>80%) occlusion increased the central venous pressure, which may exacerbate venous haemorrhage, including retrohepatic caval injury or right renal plexus injury.

In our experiment, the PV or liver parenchyma, and the SMV presented a reasonable pattern. The peak density decreased; the TTP was delayed (not valid results at 100% because of no inflow contrast); and the AUTDC linearly decreased as the occlusion degree increased. The most life-threatening complication of zone I REBOA is liver or mesenteric ischaemia, resulting in irreversible hyperlactatemia and circulatory shock. The TDC of the PV or liver indicates liver perfusion and that of SMV indicates mesenteric perfusion. As described above, the density of the aorta, IVC, or RHV was affected by aortic or caval congestion. However, the TDC of the PV and liver, or that of the SMV might reflect the degree of occlusion and organ ischaemia. In other words, we assumed that the balloon volume might indicate the perfusion of the liver and small bowel during P-REBOA. Moreover, the association of organ ischaemia and the duration of occlusion should be the subject of future investigations.

Several methods for the evaluation of blood flow or ischaemia have been reported. Microdialysis has been used to evaluate ischaemia/reperfusion injury of the intestine^22^. Regional saturation of oxygen can be measured to evaluate tissue oxygenation^23^. In a previous study, the sonographic flow was monitored during REBOA^24^. The lactate levels from the artery or SMV were measured to evaluate metabolic sequelae during P-REBOA^25^. Although each method has shown benefits in evaluating blood flow, dynamic 4D-CT is a unique technique for evaluating organ ischaemia quantitatively with minimum surgical invasion or vasospasm caused by operative procedures such as laparotomy, exposure, or dissection.

Organ ischaemia is a vital issue in the management of patients undergoing REBOA. This is the first TDC analysis in which dynamic 4D-CT was used to evaluate visceral blood flow and organ ischaemia during P-REBOA. However, this study has several limitations. First, the safety threshold of occlusion duration in each degree is still unknown. Second, this experiment did not employ haemorrhagic shock, for which REBOA is usually utilised. Third, the TDC was drawn by the average elevated density from the baseline. Thus, the individual differences and motion artefacts impede the interpretation of the results, and the value of the quantitative evaluation is limited, especially in the total REBOA (100% occlusion) settings. Fourth, the inflow location is used for TDC analysis, such as brain perfusion. However, the arterial system was not suitable to evaluate the in the aortic occlusion condition because of the interruption of the washout of the contrast, which can be a limitation of interpretation of TDCs. Fifth, this study could only evaluate the TDCs below the balloon due to the limited scan range of the dynamic 4D-CT. Despite these limitations, this study presents a preliminary but novel model of organ ischaemia. This essential scientific question cannot be addressed without the use of animals, and our experimental protocol appropriately used research animals with minimising animal suffering. Further studies will reveal the detailed relationship between the occlusion degree and organ ischaemia during P-REBOA.

## Conclusions

The dynamic 4D-CT and TDCs demonstrated blood flow and organ perfusion during P-REBOA. The TDC of the PV, SMV, and liver parenchyma may indicate liver and small-bowel perfusion. The AUTDC linearly decreased as the occlusion volume increased. The delay of the washout in the aorta and retrograde IVC flow was observed in the highly occluded status.

## Supplementary information

Supplementary information

Supplementary information 2

## References

[1] Sadeghi M (2018). The use of aortic balloon occlusion in traumatic shock: first report from the ABO trauma registry. European journal of trauma and emergency surgery: official publication of the European Trauma Society.

[2] Matsumura Y (2018). Partial occlusion, conversion from thoracotomy, undelayed but shorter occlusion: resuscitative endovascular balloon occlusion of the aorta strategy in Japan. European journal of emergency medicine: official journal of the European Society for Emergency Medicine.

[3] Brenner M (2018). Resuscitative Endovascular Balloon Occlusion of the Aorta and Resuscitative Thoracotomy in Select Patients with Hemorrhagic Shock: Early Results from the American Association for the Surgery of Trauma’s Aortic Occlusion in Resuscitation for Trauma and Acute Care Surgery Registry. Journal of the American College of Surgeons.

[4] Stannard A, Eliason JL, Rasmussen TE (2011). Resuscitative endovascular balloon occlusion of the aorta (REBOA) as an adjunct for hemorrhagic shock. The Journal of trauma.

[5] Morrison JJ (2014). The inflammatory sequelae of aortic balloon occlusion in hemorrhagic shock. The Journal of surgical research.

[6] Manzano-Nunez R (2018). A meta-analysis of the incidence of complications associated with groin access after the use of resuscitative endovascular balloon occlusion of the aorta in trauma patients. The journal of trauma and acute care surgery.

[7] Davidson AJ (2018). The pitfalls of resuscitative endovascular balloon occlusion of the aorta: Risk factors and mitigation strategies. The journal of trauma and acute care surgery.

[8] Taylor JR, Harvin JA, Martin C, Holcomb JB, Moore LJ (2017). Vascular complications from resuscitative endovascular balloon occlusion of the aorta: Life over limb?. The journal of trauma and acute care surgery.

[9] Russo RM (2016). Partial Resuscitative Endovascular Balloon Occlusion of the Aorta in Swine Model of Hemorrhagic Shock. Journal of the American College of Surgeons.

[10] Reva VA (2018). Resuscitative endovascular balloon occlusion of the aorta: what is the optimum occlusion time in an ovine model of hemorrhagic shock?. European journal of trauma and emergency surgery: official publication of the European Trauma Society.

[11] Russo, R. M. *et al*. Extending the golden hour: Partial resuscitative endovascular balloon occlusion of the aorta in a highly lethal swine liver injury model. *The journal of trauma and acute care surgery* **80**, 372-378, discussion 378–380, 10.1097/TA.0000000000000940 (2016). 10.1097/TA.0000000000000940 26670114

[12] Kuckelman, J. *et al*. Extending the Golden Hour For Zone 1 Reboa: Improved Survival and Reperfusion Injury with Intermittent Versus Continuous Reboa in a Porcine Severe Truncal Hemorrhage Model. *The journal of trauma and acute care surgery*, 10.1097/TA.0000000000001964 (2018). 10.1097/TA.0000000000001964 30080780

[13] Madurska MJ, Jansen JO, Reva VA, Mirghani M, Morrison JJ (2017). The compatibility of computed tomography scanning and partial REBOA: A large animal pilot study. The journal of trauma and acute care surgery.

[14] Adnan, S. M. *et al*. Endovascular Control of Pelvic Hemorrhage: Concomitant use of REBOA and Endovascular Intervention. *The journal of trauma and acute care surgery*, 10.1097/TA.0000000000002079 (2018). 10.1097/TA.0000000000002079 30575686

[15] Wasicek PJ (2018). Assessment of Blood Flow Patterns Distal to Aortic Occlusion Using CT in Patients with Resuscitative Endovascular Balloon Occlusion of the Aorta. Journal of the American College of Surgeons.

[16] Reva VA (2018). Defining degree of aortic occlusion for partial-REBOA: A computed tomography study on large animals. Injury.

[17] Lin L, Bivard A, Krishnamurthy V, Levi CR, Parsons MW (2016). Whole-Brain CT Perfusion to Quantify Acute Ischemic Penumbra and Core. Radiology.

[18] Chen MY (2017). Prognostic Value of Combined CT Angiography and Myocardial Perfusion Imaging versus Invasive Coronary Angiography and Nuclear Stress Perfusion Imaging in the Prediction of Major Adverse Cardiovascular Events: The CORE320 Multicenter Study. Radiology.

[19] Kim SH, Kamaya A, Willmann JK (2014). CT perfusion of the liver: principles and applications in oncology. Radiology.

[20] Tomandl BF (2003). Comprehensive imaging of ischemic stroke with multisection CT. Radiographics: a review publication of the Radiological Society of North America, Inc.

[21] Matsumura, Y. *et al*. Distal pressure monitoring and titration with percent balloon volume: feasible management of partial resuscitative endovascular balloon occlusion of the aorta (P-REBOA). *European journal of trauma and emergency surgery: official publication of the European Trauma Society*, 10.1007/s00068-019-01257-4 (2019). 10.1007/s00068-019-01257-4 31696263

[22] Strand-Amundsen RJ (2018). Ischemia/reperfusion injury in porcine intestine - Viability assessment. World journal of gastroenterology.

[23] Ookawara S (2018). Differences in tissue oxygenation and changes in total hemoglobin signal strength in the brain, liver, and lower-limb muscle during hemodialysis. Journal of artificial organs: the official journal of the Japanese Society for Artificial Organs.

[24] Hoehn MR (2019). Aortic branch vessel flow during resuscitative endovascular balloon occlusion of the aorta. The journal of trauma and acute care surgery.

[25] Sadeghi M (2018). Blood pressure targeting by partial REBOA is possible in severe hemorrhagic shock in pigs and produces less circulatory, metabolic and inflammatory sequelae than total REBOA. Injury.

