# Supplementary material for: Organ ischemia during partial resuscitative endovascular balloon occlusion of the aorta: Dynamic 4D Computed tomography in swine
Source: Sci Rep. 2020 Mar 30;10:5680. doi: 10.1038/s41598-020-62582-y (PMC7105501; doi:10.1038/s41598-020-62582-y)

Organ ischaemia during partial resuscitative endovascular balloon occlusion of the aorta: Dynamic 4D Computed tomography in swine

Yosuke Matsumura, Akiko Higashi, Yoshimitsu Izawa, Shuji Hishikawa, Hiroshi Kondo, Viktor Reva, Shigeto Oda, Junichi Matsumoto

Supplementary Materials

Supplement 1. Analyzed level of the time-density curve

Upper section included the upper aorta (1), inferior vena cava (2), right hepatic vein (3), portal vein (4), and liver parenchyma (5) were analyzed as the regions of interest. The middle section included the level on the caudal side of the two slices (6 mm) where the superior mesenteric vein joins the splenic vein was defined as the middle section. The lower section is the most distal scan range (approximately 13cm distal from the upper section) and the lower aorta was evaluated.

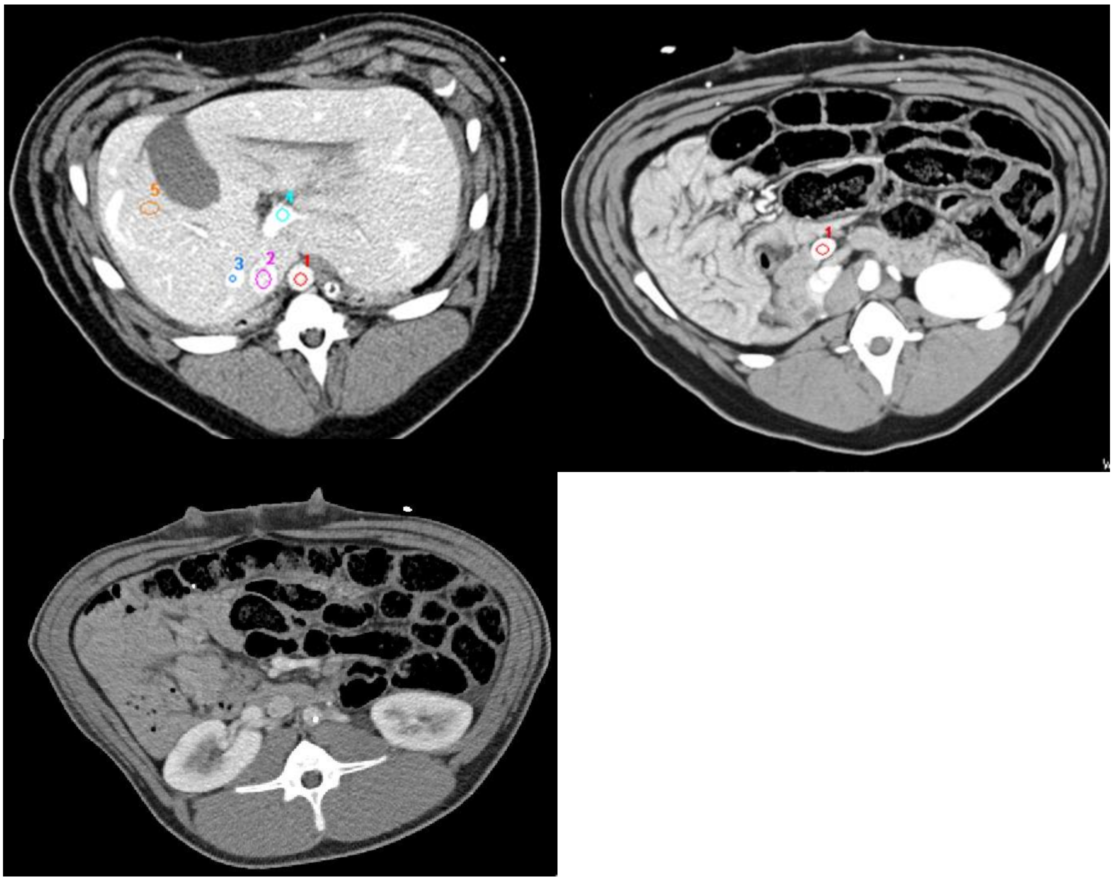

Supplement: Supplementary file 2 — Supplementary information 2 [file 41598_2020_62582_MOESM2_ESM.pdf]
